# Immunomodulation of *Lactobacillus rhamnosus* GG (LGG)-derived soluble factors on antigen-presenting cells of healthy blood donors

**DOI:** 10.1038/srep22845

**Published:** 2016-03-10

**Authors:** Fiona Long Yan Fong, Pirkka V. Kirjavainen, Hani El-Nezami

**Affiliations:** 1 School of Biological Sciences, The University of Hong Kong, HKSAR, People’s Republic of China; 2 Institute of Public Health and Clinical Nutrition, University of Eastern Finland, Kuopio, Finland.; 3 Living Environment and Health Unit, National Institute for Health and Welfare, Kuopio, Finland.

## Abstract

*Lactobacillus rhamnosus* GG (LGG) cells have been shown to promote type-1 immune responsiveness; however knowledge of immunomodulation of soluble factors secreted by LGG is limited. This is the first study to investigate whether LGG soluble factors promote a comparable immune responsiveness as the bacterial cells. Both treatments − LGG conditioned medium with (CM + LGG) or without (CM) LGG cells, in this study increased expression of several toll-like receptors (TLRs) in all studied cell types and antigen presentation-associated receptor HLA-DR in macrophages and “intermediate” monocytes; but decreased that of activation markers on monocytes and macrophages and production of IL-10, IL-12 and TNFα in macrophages. In co-culture with mononuclear cells, CM increased Th1-type cytokine profile but not as pronounced as CM + LGG. This study suggests that LGG soluble factors exert similar immunomodulatory effects as the intact cells, but cells may be required for optimal type-1 immune responsiveness polarizing capacity of this probiotic strain.

Conditioned medium of lactobacilli, which is cell-free supernatant containing soluble factors, has been shown to enhance lactobacilli but inhibit invasive *Escherichia coli*, adhere to intestinal epithelial cells, intestine mucus and gastric mucin^1^,^2^; and elicit antibacterial activity against pathogens such as *Salmonella* species^3^,^4^,^5^. Soluble factors refer to metabolites, proteins, DNA and cell-wall constituents^2^,^6^.

*Lactobacillus rhamnosus* GG (LGG) (ATCC 53103) is perhaps the most researched lactobacilli in the world; but some of its soluble factors have only been recently identified, for example, p75 and p40 proteins^7^, porcine serpine protease inhibitor, glyceraldehyde-3-phosphate dehydrogenase (GAPDH), cell wall-associated hydrolase, transcriptional regulator and phosphoglycerate kinase^8^. These factors play an important role in the physiology of LGG^8^,^9^,^10^,^11^,^12^ and have been found beneficial to the gut, for example, diminishing apoptosis of intestinal epithelium^7^,^13^, enhancing intestinal crypt survival^13^ and preserving cytoskeletal integrity of intestine^14^. However, knowledge of the immunomodulatory effects exerted by LGG soluble factors is lacking. Our previous study shows that LGG cells activate antigen-presenting cells (APCs) of healthy blood donors to promote type-1 immune responsiveness^15^. Herein, this study aimed to investigate whether LGG soluble factors promote the same immune responsiveness as the cells, and if LGG cells alter the immunomodulatory properties of the soluble factors. Elucidating the immunomodulatory effects of LGG soluble factors will help to draw evidence-based guidelines of this strain for the treatment development for various diseases such as drug synthesis and vaccine development.

## Results

### TLR mRNA expression in DCs, macrophages and monocytes

**Figure 1:**
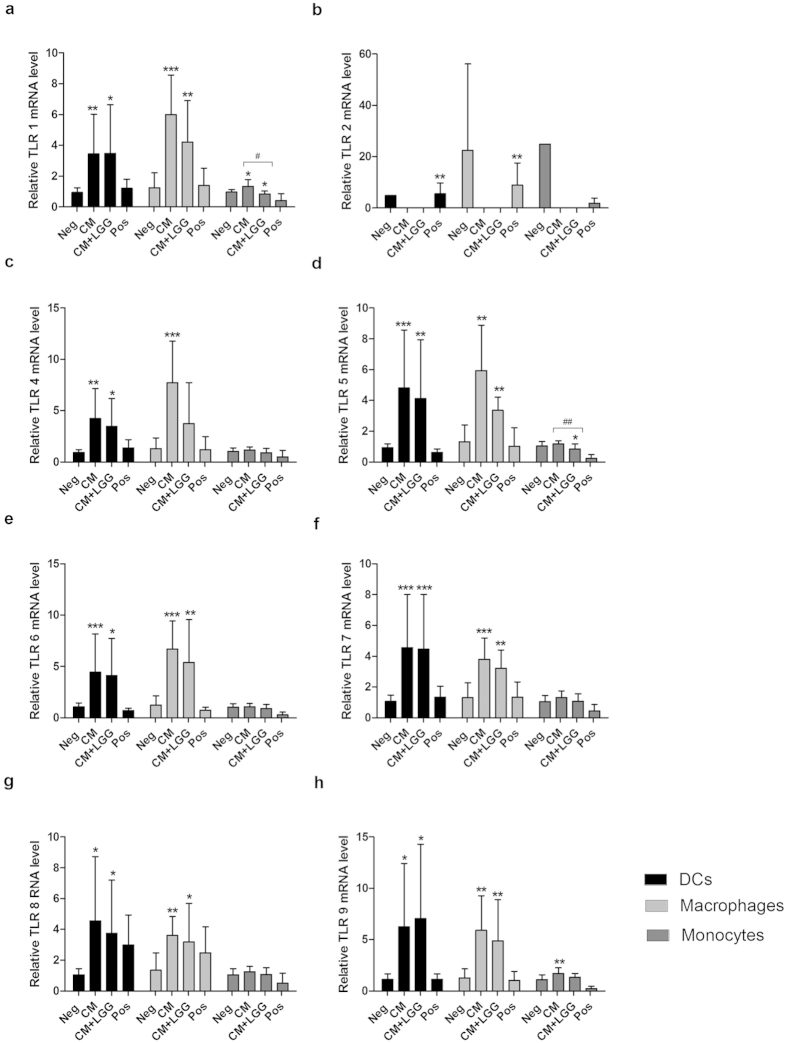
Toll-like Receptor (TLR) expression of dendritic cells (DCs), macrophages and monocytes. TLRs 1, 2, 4, 5, 6, 7, 8, and 9 expressions of monocytes, monocyte-derived DCs and macrophages of healthy blood donors (n ≥ 4) treated with LGG conditioned medium (CM); or LGG conditioned medium with LGG cells (CM + LGG); or 1000 U/ml recombinant human (rh) IFNγ, 1000 ng/ml lipopolysaccharides (LPS) and 1 μg/ml R848 (positive control; Pos); or alone (negative control; Neg) were analyzed by qPCR after 24-hour incubation. Results are presented as mean ± SD. *p < 0.05, **p < 0.01 and ***p < 0.001 in comparisons between the treated cells and corresponding negative controls. ^#^p < 0.05, ^##^p < 0.01 and ^###^p < 0.001 in comparisons between CM-treated and CM + LGG-treated cells.

TLR mRNA levels of DCs, macrophages and monocytes are shown in Fig. 1. They were detected after 24-hour incubation with CM or CM + LGG. Both CM and CM + LGG increased TLRs 1, 4, 5, 6, 7, 8 and 9 mRNA levels of DCs and macrophages. CM increased TLRs 1 and 9 mRNA levels of monocytes but CM + LGG decreased TLRs 1 and 5 mRNA levels of monocytes. Moreover, CM and CM + LGG decreased TLR 2 mRNA levels of DCs, macrophages and monocytes, which were below the detection limit. TLRs 1 and 5 mRNA levels of CM + LGG-treated monocytes were significantly lower than those of CM-treated monocytes.

### Expression of activation markers and intracellular cytokines among monocytic subsets

**Figure 2:**
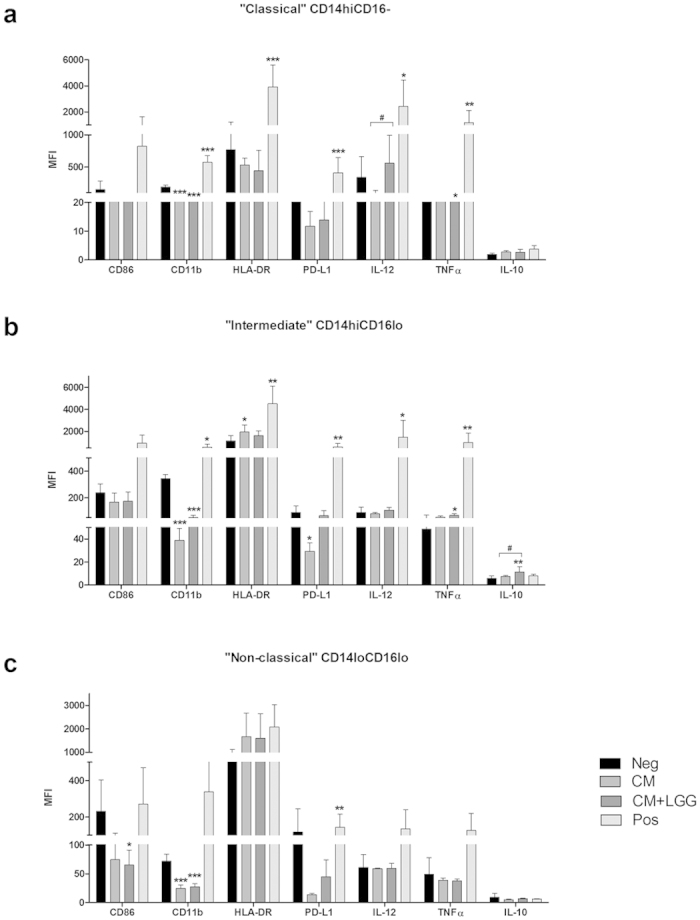
Expression of activation markers and intracellular cytokines among monocytic subsets. Mean fluorescence intensities (MFIs) of activation markers and intracellular cytokines among monocytic subsets of healthy blood donors (n ≥ 4) treated with LGG conditioned medium (CM); or LGG conditioned medium with LGG cells (CM + LGG); or 1000 U/ml recombinant human (rh) IFNγ, 1000 ng/ml lipopolysaccharides (LPS) and 1 μg/ml R848 (positive control; Pos); or alone (negative control; Neg) were determined by flow cytometer after 24-hour incubation. (**a**) “classical” CD14hiCD16-, (**b**) “intermediate” CD14hiCD16lo, and (**c**) “non-classical” CD14loCD16lo monocytic subsets were studied. Results are presented as mean ± SD. *p < 0.05, **p < 0.01 and ***p < 0.001 in comparisons between treated monocytes and negative control. ^#^p < 0.05, ^##^p < 0.01 and ^###^p < 0.001 in comparisons between CM-treated and CM + LGG-treated monocytes.

Mean Fluorescence Intensities (MFIs) of activation markers and intracellular cytokines of CM-treated or CM + LGG-treated “classical” CD14hiCD16-, “intermediate” CD14hiCD16lo and “non-classical” CD14loCD16lo monocytic subsets are shown in Fig. 2. Within “classical” CD14hiCD16- monocytic subset, both CM and CM + LGG decreased CD11b MFI but CM + LGG increased TNFα MFI. IL-12 MFI was significantly higher in CM + LGG-treated than in CM-treated monocytes. Within “intermediate” CD14hiCD16lo monocytic subset, CM decreased CD11b and PD-L1 MFIs but increased HLA-DR MFI. CM + LGG decreased CD11b MFI but increased TNFα and IL-10 MFIs. IL-10 MFI of CM + LGG-treated monocytes was significantly higher than that of CM-treated monocytes. Within “non-classical” CD14loCD16lo monocytic subset, CM + LGG significantly decreased CD86 MFI while both treatments reduced CD11b MFI.

### Expression of activation markers and intracellular cytokines in macrophages

**Figure 3:**
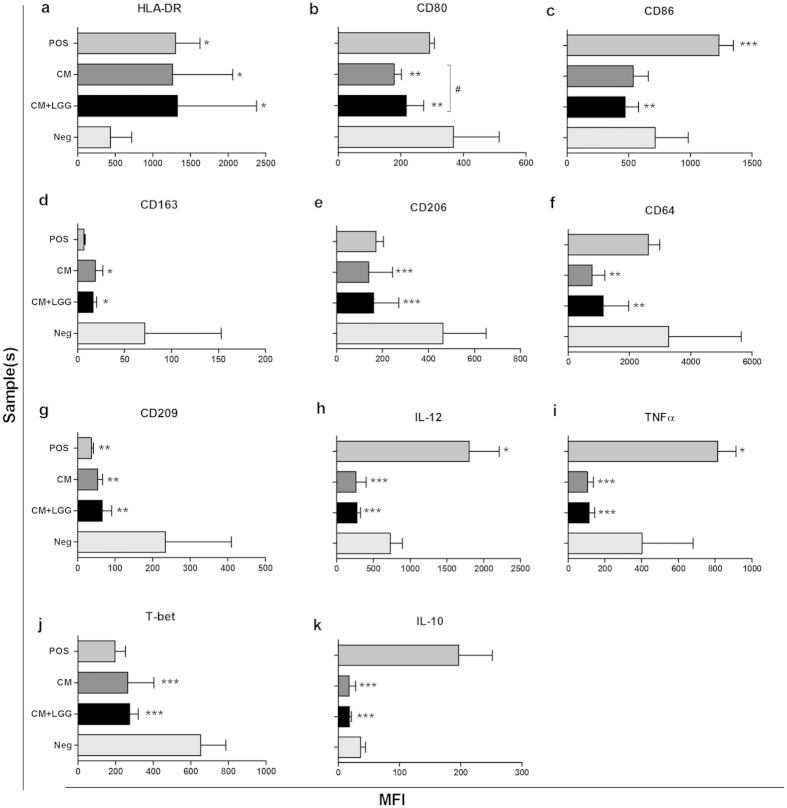
Expression of activation markers and intracellular cytokines in macrophages. MFIs of activation markers and intracellular cytokines in monocyte-derived macrophages (n ≥ 4) stimulated with LGG conditioned medium (CM); or LGG conditioned medium with LGG cells (CM + LGG); or 1000 U/ml recombinant human (rh) IFNγ, 1000 ng/ml lipopolysaccharides (LPS) and 1 μg/ml R848 (positive control; Pos); or alone (negative control; Neg) were analyzed by flow cytometer after 24-hour incubation. Results were presented as mean ± SD. *p < 0.05, **p < 0.01 and ***p < 0.001 in comparisons between treated macrophages and negative control. ^#^p < 0.05, ^##^p < 0.01 and ^###^p < 0.001 in comparisons between CM-treated and CM + LGG-treated macrophages.

MFIs of activation markers and intracellular cytokines of CM-treated or CM + LGG-treated macrophages are shown in Fig. 3. Both treatments increased HLA-DR MFI but decreased MFIs of activation markers CD80, CD86, CD163, CD206, CD64 and CD209. They also reduced MFIs of M1-type cytokines IL-12, TNFα, Th1 polarization-associated transcription factor T-bet; and M2-type cytokine IL-10. CD80 MFI of CM + LGG-treated macrophages was significantly higher than that of CM-treated macrophages.

### Cytokine secretion profiles of DC-PBMC co-cultures

**Figure 4:**
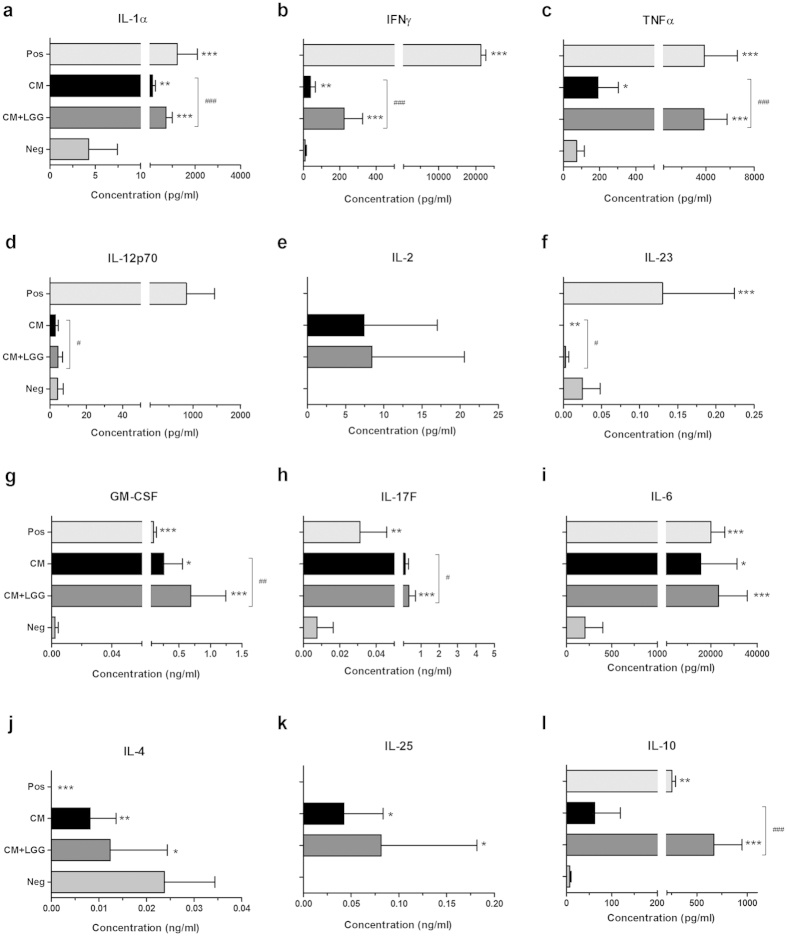
Cytokine secretion profiles of DC-peripheral blood mononuclear cell (PBMC) co-cultures. Cytokine production levels of co-cultures of autologous PBMCs and monocyte-derived DCs (n ≥ 4) pre-stimulated 24 hours with LGG conditioned medium (CM); or LGG conditioned medium with LGG cells (CM + LGG); or 1000 U/ml recombinant human (rh) IFNγ, 1000 ng/ml lipopolysaccharides (LPS) and 1 μg/ml R848 (positive control; Pos); or alone (negative control; Neg) were analyzed by multiplex assay. Results are shown as mean ± SD. *p < 0.05, **p < 0.01 and ***p < 0.001 in comparisons between treated cells and negative control. ^#^p < 0.05, ^##^p < 0.01 and ^###^p < 0.001 in comparisons between CM-treated and CM + LGG-treated cells.

Cytokine secretion profiles of co-cultures of treated DCs and mononuclear cells are shown in Fig. 4. Both treatments increased type-1 immune response-associated cytokines (IL-1α, IFNγ and TNFα) and Th17-associated cytokines (GM-CSF, IL-17F and IL-6). They decreased Th2 signature cytokine (IL-4) but increased IL-25. A pronounced increase was seen in the production of regulatory cytokine IL-10 with CM + LGG treated DCs but not with CM-treated DCs. Levels of IL-1α, IFNγ, TNFα, IL-12(p70), IL-23, GM-CSF, IL-17F and IL-10 were significantly higher in cultures with CM + LGG-treated DCs than in CM -treated DCs.

## Discussion

To the best of our knowledge, this is the first study to demonstrate the immunomodulatory effects of LGG soluble factors on APCs of human blood donors. The results indicate that the soluble factors released during LGG growth have a similar pattern but a relatively modest influence on APC activation and immunoresponse polarization as the viable bacterial cells per se.

In our previous study, we showed that LGG cells reduced TLR 2 expression of DCs, macrophages and monocytes and TLR 8 expression of macrophages of healthy blood donors^15^. In the present study, LGG soluble factors seemed to exert an opposite effect that they significantly increased TLRs 1, 4, 5, 6, 7, 8 and 9 expressions of DCs and macrophages, and TLRs 1 and 9 expressions of monocytes. Down-regulation of TLR mRNA levels has been indicated to be related to the ligation of those particular receptors^19^. In this case, the up-regulation of certain TLR expressions could be a general response due to failure of ligation of several, but not all, of the corresponding TLRs on the cell surface. The alteration of TLR expressions on the key APCs could influence the subsequent innate responsivenss to pathogen-associated molecular patterns (PAMPs)^20^,^21^. Intracellular antiviral pathways and production of antiviral effectors such as interferons (IFNs) and pro-inflammatory cytokines may be affected. Further investigations are, however, required.

One specifically interesting finding in this study is the significant reduction in the expression of several activation and inflammation markers on monocytes and macrophages, particularly the significant decrease in the expression of the adhesion molecule CD11b on all studied monocytic subsets. Increase in CD11b expression on circulating monocytes has been previously demonstrated to be associated with atherogenic inflammation and reflect the activity level of the disease^22^,^23^. Our unpublished data show that viable LGG cells (1 × 10^8^ CFU/day) singificantly suppressed the atherogenesis in ApoE−/− mice with Western-diet induced atherosclerosis (Chan *et al.*, unpublished). The treatments also consistently reduced other maturation and activation markers on the APCs but increased the expression of HLA-DR. Increase in HLA-DR expression indicates an increase in antigen presentation capacity. Antigen presentation by immature APCs theoretically promotes the formation of tolerogenic responses although no evidence has been found in the co-culture experiments^24^,^25^,^26^ with autologous mononuclear cells. Moreover, both treatments decreased the expression of Th1 polarization-associated transcription factor T-bet in macrophages, implying a decrease in the expression of another signature M1-type cytokine IFNγ^27^, which was not measured in the present study.

Instead, DCs pretreated with CM alone or CM with LGG cells significantly increased the secretions of pro-inflammatory type-1 cytokines (IL-1α, IFNγ and TNFα) in the PBMC co-cultures. While the effect was evident with both pretreatments it was significantly greater with DCs pretreated with the cocktail of CM and viable LGG cells. This implies the importance of the cellular structures of LGG over its secreted metabolic products for pro-inflammatory effects, which is supported by previous studies^28^,^29^. These studies have suggested the importance of, for example, fimbriae or pilus adhesin on LGG for adhesion, close interaction between LGG and host cells, and modulation of inflammatory responsiveness and innate immune gene expression; lipoteochoic acid (LTA) for interaction with various pattern recognition receptors (PRRs); and exopolysaccharides (EPS) for modulation of adaptation of the bacterial cells.

In summary, the present study demonstrates in an *in vitro* model of APCs that the immunomodulation of LGG conditioned growth media shows a similar pattern as that of the viable probiotic strain per se. Both treatments decreased the constitutive expressions of several TLRs in all studied APCs, and activation markers and cytokine expressions of monocytes and macrophages. Based on the cytokine secretion profile of autologous co-cultures, the treated DCs appeared to promote type-1 pro-inflammatory responsiveness; and the influence was significantly higher with than without the viable cells. All these findings are of significant relevance to *in vivo* physiological responses, for example, manipulating intestinal microbial communities; suppressing adherence of pathogens to intestines; activating anti-apoptotic genes in intestinal epithelial cells; fortifying intestinal barrier; regulating intestinal homeostasis; and modulating systemic immunity^30^,^31^,^32^,^33^.

## Conclusion

In conclusion, the soluble factors released by LGG during its growth have similar immunomodulatory effects as the intact cells, but the latter seem to be required for the optimal type-1 immune responsiveness polarizing capacity of this probiotic strain. These results can provide an evidence-based insight for further clinical development of this strain, such as drug synthesis and vaccine development, for various diseases.

## Methods

### Derivation of dendritic cells and macrophages from monocytes

Human peripheral blood mononuclear cells (PBMCs) were isolated from healthy blood donors (n ≥ 4) (Hong Kong Red Cross Blood Transfusion Service, Hong Kong, China) by density gradient centrifugation over Ficoll-Plaque^TM^ Plus (GE Healthcare Life Sciences, Piscataway, NJ, USA) and seeded in complete RPMI-1640 medium (LONZA, Basel, Switzerland) for 2 hours at 37 °C to isolate monocytes. Extra PBMCs were frozen for later experiments. Isolated monocytes were derived to immature DCs in the presence of 40 ng/ml recombinant human (rh) granulocyte-macrophage colony-stimulating factor (GM-CSF) and 40 ng/ml rh interleukin (IL)-4 (both from PeproTech EC. Ltd, London, UK); or to immature macrophages in the presence of 40 ng/ml rh GM-CSF. Cells were incubated for 7 days with fresh supplemented medium added every 2 days. Protocol was modified from the studies of Lacey (2012) and Mohamadzadeh (2005)^16^,^17^, and approved by the University of Hong Kong. All methods were carried out in accordance with the approved guidelines.

### Bacterial strain and stimulation of antigen-presenting cells (APCs)

LGG (ATCC 53103) was cultivated in De Man, Rogosa, Sharpe (MRS) broth (LAB M Limited, Lancashire, UK) anaerobically at 37 °C to logarithm-phase. Cell-free conditioned medium (CM) was prepared by centrifugation at 3000 rpm for 15 minutes at 4 °C and dual filtration through 0.2 μm Acrodisc Syringe Filters (Life Sciences, MI, USA). Immature DCs, macrophages and monocytes were stimulated with CM or CM in the presence of LGG (CM + LGG) for 24 hours.

### Detection of toll-like receptor (TLR) expression by quantitative real-time PCR (qPCR)

TLRs 1, 2, 4, 5, 6, 7, 8 and 9 mRNA levels of DCs, macrophages and monocytes were determined by qPCR with modified protocol and sequences of primers and probes from the study of Flacher *et al.*^18^. Data were acquired on ABI StepOne Plus Real-Time PCR System (Life Technologies, NY, USA).

### Detection of expressions of activation markers and intracellular cytokines of monocytes and macrophages by flow cytometry

Brefeldin A solution (eBioscience, CA, USA) was added to monocytes and macrophages cultures (10 μg/ml) 15 hours before the cells were harvested. Cells were stained with appropriate anti-human monoclonal antibodies such as CD14-FITC (eBioscience), CD16-BD Horizon PE-CF594 (BD Biosciences, New Jersey, USA), CD86-PE (eBioscience), CD11b-PE/Cy7 (eBioscience), HLA-DR-APC/Cy7 (BioLegend, CA, USA), PD-L1-APC (eBioscience), CD80-PerCP3Flour^®^710 (eBioscience), CD163-APC (eBioscience), CD206-APC/Cy7 (Biolegend), CD64-PE (Biolegend), CD209-PE/Cy7 (eBioscience), T-bet-PerCP/Cy5.5 (eBioscience), IL-12(p40)-PE (eBioscience), IL-10-Alexa Fluor 647 (eBioscience) and TNFα-PerCP-Cy5.5 (BD Pharmingen^TM^) following manufacturer’s protocol. Data were acquired on FACSAria III flow cytometer (BD Biosciences) and analyzed with FlowJo Version 7.6 (Ashland, OR, USA) based on 20,000 cells.

### Detection of cytokine levels by multiplex assay

Treated DCs were washed with PBS and incubated with autologous PBMCs in a 1:20 ratio for 3 days. Supernatant was collected and stored at −20 °C for cytokine detection. Levels of IL-1α, IFNγ, TNFα, IL-12(p70), IL-2, IL-23, GM-CSF, IL-17F, IL-6, IL-4, IL-25 and IL-10 were determined by Luminex^®^ 200^TM^ with xPONENT 3.1 software (Luminex Corporation, Texas, USA) following manufacturer’s protocol and analyzed by MILLIPLEX Analyst Version 3.5.5.0 (Vigene Tech Inc., Carlisle, MA, USA).

### Statistical Analysis

Results were shown as mean ± standard deviation (SD) and analyzed with Kruskal-Wallis test, Mann-Whitney test, analysis of variance and two-tailed Student’s t test. CM-treated and CM + LGG-treated cells were compared with the negative control and with each other. P-values below 0.05 were considered as significant. Statistical calculations were performed using GraphPad Software Prism Version 6.04 (San Diego, CA, USA) and SPSS Version 19 for Windows (Chicago, IL, USA).

## Additional Information

**How to cite this article**: Fong, F. L. Y. *et al.* Immunomodulation of *Lactobacillus rhamnosus* GG (LGG)-derived soluble factors on antigen-presenting cells of healthy blood donors. *Sci. Rep.* **6**, 22845; doi: 10.1038/srep22845 (2016).

## References

[b1] Ingrassia I., Leplingard A. & Darfeuille-Michaud A. Lactobacillus casei DN-114 001 inhibits the ability of adherent-invasive *Escherichia coli* isolated from Crohn’s disease patients to adhere to and to invade intestinal epithelial cells. Appl Environ Microbiol 71, 2880–2887, 10.1128/AEM.71.6.2880-2887.2005 (2005). 15932981 PMC1151832

[b2] Rojas M., Ascencio F. & Conway P. L. Purification and characterization of a surface protein from Lactobacillus fermentum 104R that binds to porcine small intestinal mucus and gastric mucin. Appl Environ Microbiol 68, 2330–2336 (2002). 11976105 10.1128/AEM.68.5.2330-2336.2002 PMC127527

[b3] Bernet-Camard M. F. *et al.* The human Lactobacillus acidophilus strain LA1 secretes a nonbacteriocin antibacterial substance(s) active *in vitro* and *in vivo*. Appl Environ Microbiol 63, 2747–2753 (1997). 9212421 10.1128/aem.63.7.2747-2753.1997 PMC168570

[b4] Coconnier M. H., Lievin V., Bernet-Camard M. F., Hudault S. & Servin A. L. Antibacterial effect of the adhering human Lactobacillus acidophilus strain LB. Antimicrob Agents Chemother 41, 1046–1052 (1997). 9145867 10.1128/aac.41.5.1046 PMC163848

[b5] Fayol-Messaoudi D., Berger C. N., Coconnier-Polter M. H., Lievin-Le Moal V. & Servin A. L. pH-, Lactic acid-, and non-lactic acid-dependent activities of probiotic Lactobacilli against Salmonella enterica Serovar Typhimurium. Appl Environ Microbiol 71, 6008–6013, 10.1128/AEM.71.10.6008-6013.2005 (2005). 16204515 PMC1266002

[b6] Mirnejad R., Vahdati A. R., Rashidiani J., Erfani M. & Piranfar V. The antimicrobial effect of lactobacillus casei culture supernatant against multiple drug resistant clinical isolates of Shigella sonnei and Shigella flexneri *in vitro*. Iranian Red Crescent medical journal 15, 122–126, 10.5812/ircmj.7454 (2013). 23682323 PMC3652498

[b7] Yan F. *et al.* Soluble proteins produced by probiotic bacteria regulate intestinal epithelial cell survival and growth. Gastroenterology 132, 562–575, 10.1053/j.gastro.2006.11.022 (2007). 17258729 PMC3036990

[b8] Sanchez B., Schmitter J. M. & Urdaci M. C. Identification of novel proteins secreted by *Lactobacillus rhamnosus* GG grown in de Mann-Rogosa-Sharpe broth. Lett Appl Microbiol 48, 618–622, 10.1111/j.1472-765X.2009.02579.x (2009). 19416463

[b9] Kinoshita H. *et al.* Cell surface Lactobacillus plantarum LA 318 glyceraldehyde-3-phosphate dehydrogenase (GAPDH) adheres to human colonic mucin. J Appl Microbiol 104, 1667–1674, 10.1111/j.1365-2672.2007.03679.x (2008). 18194256

[b10] Kinoshita H. *et al.* Cell surface glyceraldehyde-3-phosphate dehydrogenase (GAPDH) of Lactobacillus plantarum LA 318 recognizes human A and B blood group antigens. Research in microbiology 159, 685–691, 10.1016/j.resmic.2008.07.005 (2008). 18790050

[b11] Chatfield C. H., Koo H. & Quivey R. G. Jr. The putative autolysin regulator LytR in Streptococcus mutans plays a role in cell division and is growth-phase regulated. Microbiology 151, 625–631, 10.1099/mic.0.27604-0 (2005). 15699211

[b12] Hathaway L. J., Battig P. & Muhlemann K. *In vitro* expression of the first capsule gene of Streptococcus pneumoniae, cpsA, is associated with serotype-specific colonization prevalence and invasiveness. Microbiology 153, 2465–2471, 10.1099/mic.0.2006/005066-0 (2007). 17660411

[b13] Ciorba M. A. *et al.* Lactobacillus probiotic protects intestinal epithelium from radiation injury in a TLR-2/cyclo-oxygenase-2-dependent manner. Gut 61, 829–838, 10.1136/gutjnl-2011-300367 (2012). 22027478 PMC3345937

[b14] Tao Y. *et al.* Soluble factors from Lactobacillus GG activate MAPKs and induce cytoprotective heat shock proteins in intestinal epithelial cells. Am J Physiol Cell Physiol 290, C1018–1030, 10.1152/ajpcell.00131.2005 (2006). 16306130

[b15] Fong F. L. Y., Kirjavainen P., Wong V. H. Y. & El-Nezami H. Immunomodulatory effects of *Lactobacillus rhamnosus* GG on dendritic cells, macrophages and monocytes from healthy donors. Journal of Functional Foods 13, 71–79, 10.1016/j.jff.2014.12.040 (2015).

[b16] Lacey D. C. *et al.* Defining GM-CSF- and macrophage-CSF-dependent macrophage responses by *in vitro* models. J Immunol 188, 5752–5765, 10.4049/jimmunol.1103426 (2012). 22547697

[b17] Mohamadzadeh M. *et al.* Lactobacilli activate human dendritic cells that skew T cells toward T helper 1 polarization. Proc Natl Acad Sci USA 102, 2880–2885, 10.1073/pnas.0500098102 (2005). 15710900 PMC549474

[b18] Flacher V. *et al.* Human Langerhans cells express a specific TLR profile and differentially respond to viruses and Gram-positive bacteria. J Immunol 177, 7959–7967 (2006). 17114468 10.4049/jimmunol.177.11.7959

[b19] Peroval M. Y., Boyd A. C., Young J. R. & Smith A. L. A critical role for MAPK signalling pathways in the transcriptional regulation of toll like receptors. PLoS One 8, e51243, 10.1371/journal.pone.0051243 (2013). 23405061 PMC3566169

[b20] Yu S. L. *et al.* Antagonist-mediated down-regulation of Toll-like receptors increases the prevalence of human papillomavirus infection in systemic lupus erythematosus. Arthritis Res Ther 14, R80, 10.1186/ar3803 (2012). 22513098 PMC3446454

[b21] Zhang E. & Lu M. Toll-like receptor (TLR)-mediated innate immune responses in the control of hepatitis B virus (HBV) infection. Med Microbiol Immunol 204, 11–20, 10.1007/s00430-014-0370-1 (2015). 25550115 PMC4305100

[b22] Meisel S. R. *et al.* Increased expression of neutrophil and monocyte adhesion molecules LFA-1 and Mac-1 and their ligand ICAM-1 and VLA-4 throughout the acute phase of myocardial infarction: possible implications for leukocyte aggregation and microvascular plugging. J Am Coll of Cardiol 31, 120–125 (1998). 9426029 10.1016/s0735-1097(97)00424-5

[b23] Woollard K. J. & Geissmann F. Monocytes in atherosclerosis: subsets and functions. Nat Rev Cardiol 7, 77–86, 10.1038/nrcardio.2009.228 (2010). 20065951 PMC2813241

[b24] Manicassamy S. & Pulendran B. Dendritic cell control of tolerogenic responses. Immunol Rev 241, 206–227, 10.1111/j.1600-065X.2011.01015.x (2011). 21488899 PMC3094730

[b25] Steinman R. M., Hawiger D. & Nussenzweig M. C. Tolerogenic dendritic cells. Annu Rev Immunol 21, 685–711, 10.1146/annurev.immunol.21.120601.141040 (2003). 12615891

[b26] Steinman R. M. & Nussenzweig M. C. Avoiding horror autotoxicus: the importance of dendritic cells in peripheral T cell tolerance. Proc Natl Acad Sci USA 99, 351–358, 10.1073/pnas.231606698 (2002). 11773639 PMC117564

[b27] Lighvani A. A. *et al.* T-bet is rapidly induced by interferon-gamma in lymphoid and myeloid cells. Proc Natl Acad Sci USA 98, 15137–15142, 10.1073/pnas.261570598 (2001). 11752460 PMC64996

[b28] Segers M. E. & Lebeer S. Towards a better understanding of *Lactobacillus rhamnosus* GG–host interactions. Microbial cell factories 13 Suppl 1, S7, 10.1186/1475-2859-13-S1-S7 (2014). 25186587 PMC4155824

[b29] Ganguli K. *et al.* *Lactobacillus rhamnosus* GG and its SpaC pilus adhesin modulate inflammatory responsiveness and TLR-related gene expression in the fetal human gut. Pediatr Res 77, 528–535, 10.1038/pr.2015.5 (2015). 25580735 PMC4465787

[b30] Fong F. L. Y., Shah N. P., Kirjavainen P. & El-Nezami H. Mechanism of action of probiotic bacteria on intestinal and systemic immunities and antigen-presenting cells. International Reviews of Immunology 25, 1–11 (2015). 10.3109/08830185.2015.1096937 26606641

[b31] van Baarlen P., Wells J. M. & Kleerebezem M. Regulation of intestinal homeostasis and immunity with probiotic lactobacilli. Trends Immunol 34, 208–215, 10.1016/j.it.2013.01.005 (2013). 23485516

[b32] Ganesh B. P. & Versalovic J. Luminal Conversion and Immunoregulation by Probiotics. Front Pharmacol 6, 269, 10.3389/fphar.2015.00269 (2015). 26617521 PMC4641912

[b33] Thomas C. M. & Versalovic J. Probiotics-host communication: Modulation of signaling pathways in the intestine. Gut Microbes 1, 1–16 (2010). 20672012 10.4161/gmic.1.3.11712 PMC2909492

